# Temporal analysis of cigarette butt accumulation on a touristic beach in Cartagena, Colombia

**DOI:** 10.1007/s11356-025-36752-2

**Published:** 2025-08-06

**Authors:** Claudia Díaz-Mendoza, Javier Mouthon-Bello, Camilo M. Botero, Juan Valdelamar Villegas, Leonardo Gutiérrez

**Affiliations:** 1 Universidad Tecnologica de Bolívar, Cartagena, Colombia; 2 Faculty of Engineering of the University of Cartagena, Cartagena, Colombia; 3 Joaquín Aarón Manjarrez Research Group, Sergio Arboleda University, Santa Marta, Colombia; 4 Faculty of Engineering, Fundación Universitaria Tecnológico Comfenalco, Cartagena, Colombia; 5 Particle and Interfacial Technology Group, Department of Green Chemistry and Technology, Faculty of Bioscience Engineering, Ghent University, Ghent, Belgium; 6 Facultad Del Mar y Medio Ambiente, Universidad Del Pacifico, Guayaquil, Ecuador

**Keywords:** Environmental indicators, Coastal pollution, Cigarette butts, Beach, Litter density

## Abstract

Cigarette butts (CBs) are among the most common types of litter found in coastal environments, including beach sand. They are considered hazardous waste due to their significant toxicity and the potential threats they pose to both ecosystems and public health. This study examines historical CB density data collected from Bocagrande Beach, Colombia, across two distinct periods: 2011–2015 and 2021–2022. It includes an assessment of variations in key pollution indexes, the Plastic Abundance Index (PAI), Clean Coast Index (CCI), Cigarette Butt Abundance Index (CBAI), and Cigarette Butts Pollution Index (CBPI). The methodology used visual identification surveys and the systematic collection of CBs and cigarette butt fibers (CBFs) within 500-m^2^ transects. A key finding of this research was the average solid waste density between 2011 and 2015 of 0.63 items/m^2^, with CBs being the most prevalent at 0.19 items/m^2^. Remarkably, an average of 0.6 CBs/m^2^ was observed in the 2021–2022 period, demonstrating an increasing trend in cigarette butt pollution. The CBPI values ranged from 2.28 to 31.16, classifying the beach from polluted to severely polluted conditions.

**Supplementary Information:**

The online version contains supplementary material available at 10.1007/s11356-025-36752-2.

## Introduction

Marine litter refers to human-made materials discarded or abandoned, eventually entering coastal and marine ecosystems. These debris includes a wide range of items, from household and industrial waste to lost fishing gear, recreational equipment, and, most commonly, plastic fragments (Hassan et al. 2024). Beach litter, widely recognized as a key indicator of marine pollution (Calderisi et al. 2024), poses severe risks to marine life, coastal habitats, human health, and economic activities (Cesarano et al. 2023). The increasing intensity of human activities and coastal development has exacerbated beach litter, causing significant harm to both terrestrial and marine ecosystems. Once deposited on beaches, beach litter often migrates into the marine environment, disrupting marine organisms and their habitats (Barik et al. 2024).

Urban beaches exhibit a significantly higher load of plastic litter compared to non-urban beaches. This differential impact is particularly reflected in the greater presence of CBs, rigid plastics, and foamed plastic materials, highlighting a more intense anthropogenic pressure on urbanized coastal environments (Heard 2024). Notably, beaches with lower population density and greater distance from urban centers exhibit reduced CB accumulation, suggesting that human activity is a key driver of CB contamination (Jokar et al. 2024). The Eastern Pacific (EP) region, and in particular its central and southern sectors, has received comparatively less scientific attention regarding marine debris contamination than other global regions. Recent studies have documented the occurrence of cigarette butts (CBs) along the continental coastlines of the southern EP, with sparse reports from the northern continental EP and offshore locations, and virtually no detection at the sea surface across both coastal and oceanic environments. This distribution pattern is likely due to the low buoyancy or short-term floatability of CBs, indicating a predominantly local, land-based origin for these pollutants (Honorato-Zimmer et al. 2024).

Cigarette butts are classified as hazardous waste due to their potential to cause direct or indirect harm to both human health and wildlife. Their toxicological effects on aquatic organisms are increasingly evident, particularly in calcifying species. Foraminifera exposed to CBs exhibit significant physiological stress, including shell decalcification and disrupted biomineralization, linked to the absorption of up to 85% of synthetic nicotine present in the leachate (Sabbatini et al. 2023). Acute toxicity assays have demonstrated lethal outcomes in benthic foraminifera at concentrations as low as 1 CB/L, with increased mortality and shell degradation observed from 4 CBs/L, effects primarily associated with acidification and the release of toxic compounds such as nicotine (Caridi et al. 2020). In freshwater systems, *Chironomus riparius* larvae exposed to sediments containing both smoked and unsmoked CBs showed over 20% higher mortality, reduced growth, and more than 80% inhibition in development, underscoring the teratogenic and ecotoxicological risks posed by CBs to aquatic invertebrates (Nitschke et al. 2023).

This study analyzes data collected from two monitoring campaigns conducted on Bocagrande Beach in Cartagena, Colombia, selected as a pilot site due to its high tourist activity. The first campaign was carried out monthly between 2011 and 2015, focusing on the abundance and density of solid waste discarded on the sand. Its findings identified CBs as the most prevalent litter item, prompting a second monthly campaign between 2021 and 2022 that specifically assessed the abundance and density of CBs and their fibers (CBFs). Key results include a marked increase in CB density over time and the calculation of the Cigarette Butt Pollution Index (CBPI), which classified the beach as severely contaminated. These findings contribute to the growing body of evidence from other coastal areas, such as those in Ecuador, Brazil, Argentina, Mexico, Bangladesh, Vietnam, and Spain, reporting similar levels of pollution. The insights gained may inform the development of targeted strategies for controlling and mitigating the environmental impacts of CBs and CBFs on tourist beaches.

## Materials and methods

The following methodological sections provide a detailed description of the procedures used for data collection and analysis during the two monitoring campaigns, enabling a comparative assessment of cigarette butt pollution across two distinct time periods on the same beach.

### Beach and study zones

Bocagrande Beach, located in Cartagena, Colombia, was selected as the study site. Its geographic coordinates are 10°28′56.7″ N and 75°33′42.0″ W. Cartagena has a humid tropical climate characterized by average annual temperatures of around 28 °C and an average monthly precipitation of 20.2 mm, within an area of 85.72 km^2^. The region experiences three main seasons: strong winds and low runoff (January–April), weak winds and moderate runoff (May–August), and weak winds with high runoff (September–December) (Neckel et al. 2024).

Bocagrande Beach is characterized as urban and has a dissipative profile (Cueto Fonseca et al. 2021), featuring fine sands with grain sizes ranging from 0.08 to 0.42 mm, sandy beaches that are dynamic and subject to erosion and sediment transport due to wave action (Cueto and Otero 2020). The beach is influenced by both natural sediment transport and human activities, which affect water quality and sediment distribution (Eljaiek-Urzola et al. 2023). Bocagrande is a coastal area with a low elevation, which makes it susceptible to flooding, particularly during high tides and storm surges (Van Miltenburg et al. 2015).

Cartagena is a city with significant historical and tourist appeal, and it also serves as the site of the main commercial port and a wide range of industries. Historically, it has been impacted by various environmental issues and severe pollution (Romero-Murillo et al. 2023). Bocagrande Beach has been studied and reported to have a high abundance of waste, with discarded cigarette butts being particularly prevalent on the sand (Díaz-Mendoza et al. 2023).

**Fig. 1:**
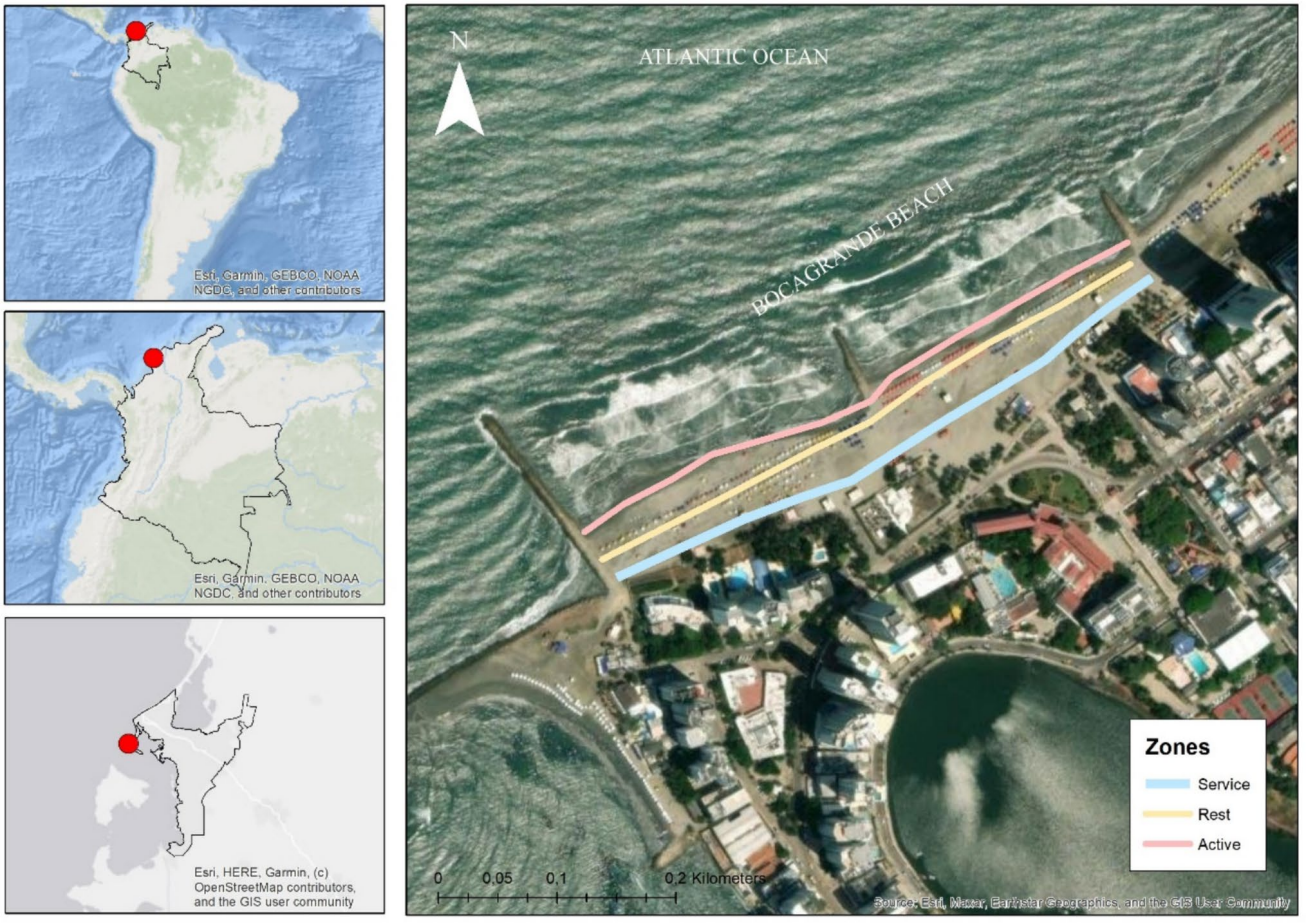
Map showing the location of monitoring zones on Bocagrande Beach, Cartagena, Colombia. The colored lines represent different functional zones based on beach use: blue indicates the service zone, yellow corresponds to the resting zone, and pink represents the active zone

Three study zones were selected based on the activities performed in each of them. The following considerations were applied to the project, according to Valdemoro and Jiménez (2006). (A) Active zone: designated for sports and recreational activities such as walking, jogging, beach soccer, and beach volleyball. This zone allows entry to the bathing area. (B) Rest zone: designated for relaxation and rest for visitors. This zone is equipped with umbrellas, chairs, and other services. (C) Service zone: an area where tourist and support services such as bars, restaurants, and souvenir shops are located (Fig. 1).

### First monitoring campaign (2011–2015)

During this time, monthly monitoring took place from February to November (the sampling was conducted on the third Sunday of each month, with data collection at 10:00 am and 4:00 pm), measuring the quantity of solid waste within 100-m^2^ transects positioned in active, rest, and service zones (the litter collection methodology applied in the active zone was based on the approach proposed by Williams et al. 2016 and is illustrated in Supplementary Fig. 1). The waste categories included paper and cardboard, glass, metals, textiles, expanded polystyrene (Styrofoam), wood, plastics, organics (food scraps), construction and demolition waste (CDW), and cigarette butts (CBs).

### Second monitoring campaign (2021–2022)

**Fig. 2:**
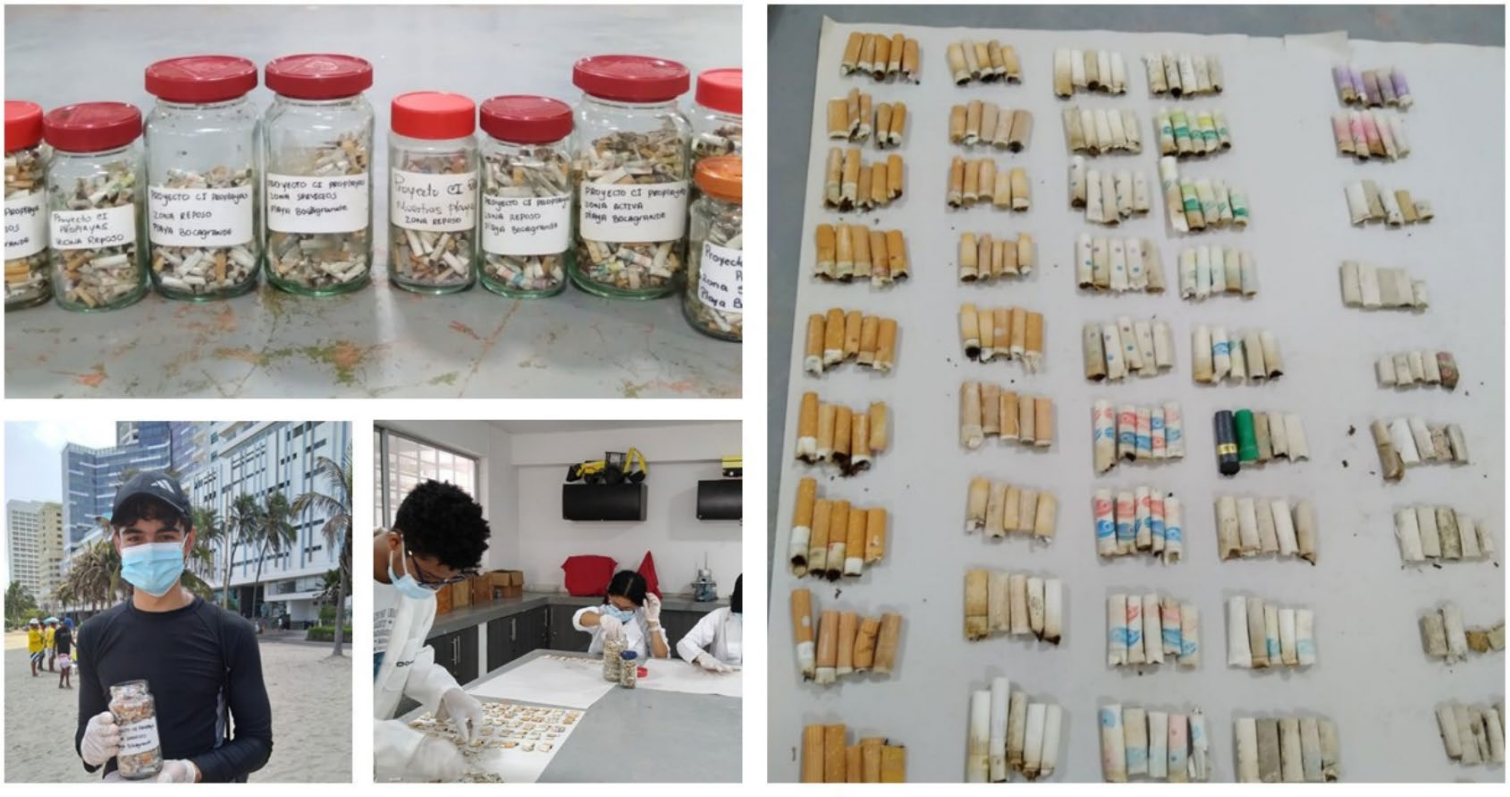
Sampling and quantification processes of CBs and CBFs

The second monitoring campaign was carried out monthly from June 2021 to December 2022, with a specific focus on CBs and CBFs, following the findings of the first campaign, which identified CBs as the most abundant waste item on Bocagrande Beach. Samples were collected on the last Sunday of each month at 9:00 am using a standardized protocol. In each predefined zone, delineated according to beach use, 500-m-long and 1-m-wide transects were surveyed, with visual identification of litter items conducted in accordance with OSPAR guidelines (Yousefi Nasab et al. 2022). To ensure comprehensive data collection, additional considerations included beach accessibility, visitor density, prevailing environmental conditions, and contextual factors such as recent meteorological or oceanographic events, as well as social activities held on the beach in the hours prior to sampling. The collected CBs and CBFs were stored separately in reusable glass jars and transported to the laboratory for subsequent weighing and unit counting according to their respective collection zones (Fig. 2).

### Assessment of contamination indexes on Bocagrande Beach

This study applied four quantitative indexes to assess the abundance and impact of litter, specifically cigarette butts (CBs), on Bocagrande Beach.

The Clean Coast Index (CCI) was calculated following the method proposed by Alkalay et al. (2007), which assesses beach cleanliness using plastic waste as an indicator. A modified version by Rangel-Buitrago et al. (2020) was adopted, relating CCI to the total amount of litter on the sand to facilitate comparison with other beaches in the Colombian Caribbean. The CCI is expressed as

1 $$CCI=\frac{\Sigma litter\;items}{Length\left(m\right)\ast Width(m)}\ast K(20)$$

where *K* = 20 for coastal areas. CCI values categorize beach cleanliness as follows: very clean (0–2), clean (2.1–5), moderate (5.1–10), dirty (10.1–20), and extremely dirty (> 20).

The Plastic Abundance Index (PAI), as defined by Rangel-Buitrago et al. (2021), quantifies plastic presence relative to total litter:

2 $$PAI=\frac{\frac{\sum plastic\;litter}{Log10\sum total\;litter\;items}}{Area}\times20$$

PAI values are interpreted as low (0.1–1), moderate (1.1–4), high (4.1–8), and very high abundance (> 8).

Based on the PAI, the Cigarette Butt Abundance Index (CBAI) was calculated to assess CB presence:

3 $$CBAI=\frac{\frac{\sum CB\;litter}{Log10\sum total\;litter\;items}}{Area}\times20$$

CBAI values follow the same interpretation scale as PAI, indicating levels of CB abundance.

Lastly, the Cigarette Butt Pollution Index (CBPI) was computed following Torkashvand et al. (2021) to evaluate the environmental impact of CBs, considering factors like persistence, contaminant release, and cleanup challenges:

4 $$CBPI=density\;o\;fcigarette\;butts\ast E$$

where *E* = 20 for sandy coastal environments. CBPI values indicate pollution levels as follows: very low (≤ 1), low (1.1–2.5), moderate (2.6–5), significant (5.1–7.5), high (7.6–10), and severe (> 10).

### Statistical analysis

Descriptive statistics were calculated to summarize the data, including measures of central tendency (arithmetic mean) and dispersion (standard deviation). To assess differences in the variables length, weight, and density of CBs and CBFs, as well as the Cigarette Butt Pollution Index (CBPI), a one-way ANOVA was performed. Tukey’s post hoc test was applied when the assumptions of normality, homogeneity of variances, and independence of residuals were satisfied. In cases where these assumptions were not met, the non-parametric Kruskal–Wallis test was used as an alternative. A significance level of 0.05 was established for all comparisons. Statistical analyses and graphical representations were conducted using GraphPad Prism 8.0 (GraphPad Software, Boston, MA, USA) and R version 4.3.1 (packages: Rcmdr and ggplot2) (R Core Team, (R 2021).

## Results and discussion

### Assessment of beach litter data from the 2011–2015 monitoring period at the study site

During the 2011–2015 monitoring period, plastics and CBs were identified as the most abundant litter types on Bocagrande Beach (Supplementary Fig. 2). The average density of beach litter recorded during the study was 0.63 items/m^2^, with plastics accounting for 0.16 items/m^2^ and CBs for 0.19 items/m^2^. Plastics exhibited a notable increasing trend over the 5-year period, rising from 21 to 34% of the total litter composition. In contrast, CBs consistently represented more than 20% of the litter, reaching a peak contribution of 48% in 2011. Other waste categories, including textiles, expanded polystyrene (Styrofoam), and construction and demolition waste (CDW), were found in smaller proportions, each ranging between 1 and 3% of the total litter collected.

There is worldwide interest in understanding the density of litter present in the sand, as well as its categories. In Vietnam, for example, extensive marine plastic pollution is evident; the abundance, type, and origin of plastic waste were examined on seven beaches along the coast of Nha Trang, Vietnam, showing average plastic abundance results on the order of 19.8 ± 19.5 items/m^2^ (Fruergaard et al. 2023). In the Moroccan beaches of the Mediterranean, an average abundance of 0.58 items/m^2^ was reported, with the majority of waste composed of human-origin polymeric materials, among which plastics constitute the majority of the items collected, mainly including caps, food wrappers, plastic/polystyrene, CBs, and small plastic bags (Bouzekry et al. 2022).

In this context, it is important to recognize that litter accumulation on beaches is influenced not only by environmental factors and marine currents but also by the number of beachgoers. Visitor presence is largely determined by weather conditions, with sunny days and peak tourist seasons typically associated with higher litter volumes. In contrast, adverse weather conditions, such as wind, rain, or overcast skies, tend to reduce beach attendance and, consequently, the amount of waste generated (Fernández García et al. 2024). Understanding these dynamics is essential for accurately interpreting variations in litter density and for developing effective beach cleanliness management strategies.

**Fig. 3:**
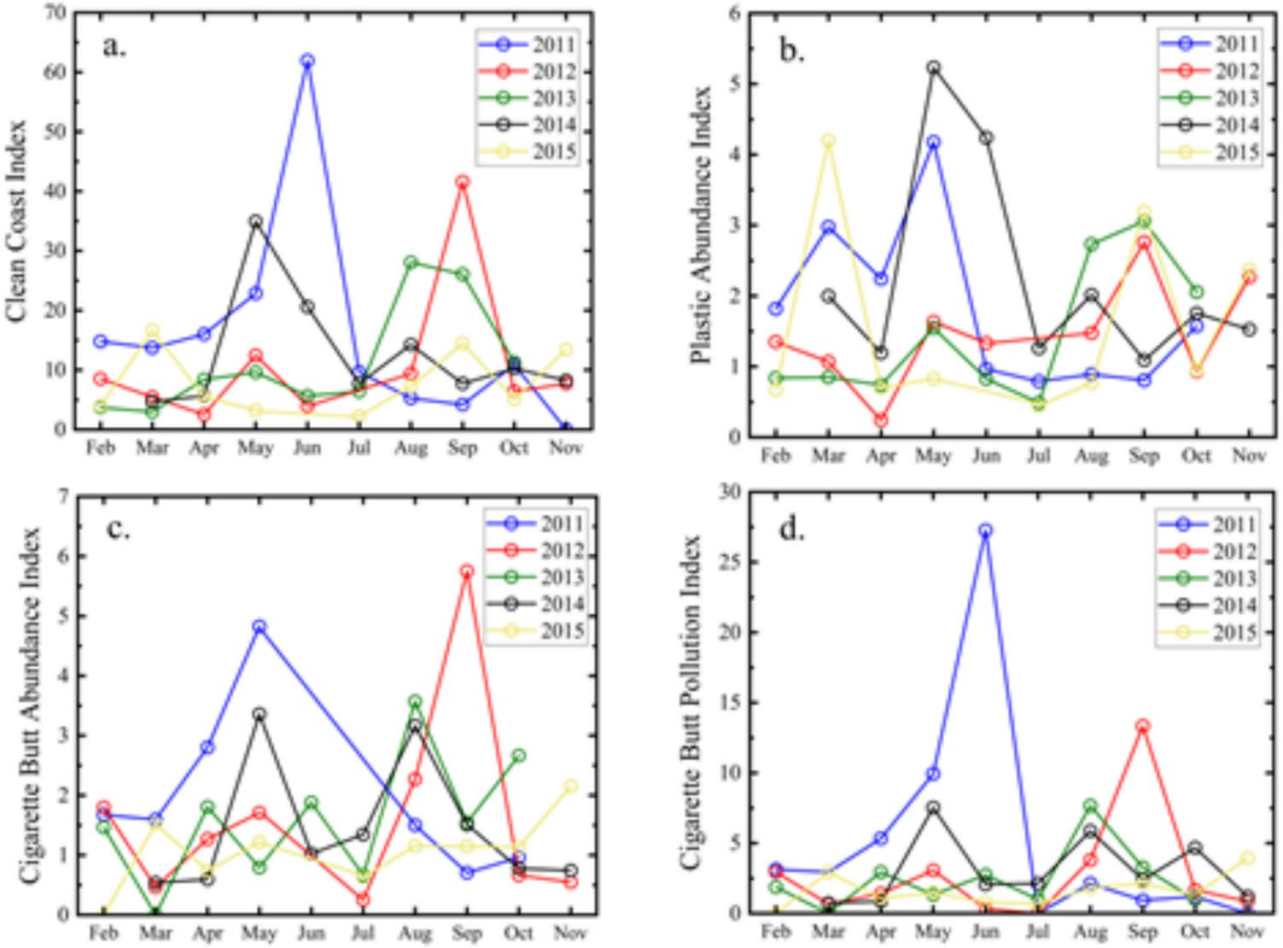
Contamination indexes calculated for Bocagrande Beach, Cartagena (2011–2015). **a** Clean Coast Index: very clean (0–2), clean (2.1–5), moderate (5.1–10), dirty (10.1–20), and extremely dirty (> 20). **b** Plastic Abundance Index: low (0.1–1), moderate (1.1–4), high (4.1–8), and very high abundance (> 8). **c** Cigarette Butt Abundance Index: low (0.1–1), moderate (1.1–4), high (4.1–8), and very high abundance (> 8). **d** Cigarette Butt Pollution Index: very low (≤ 1), low (1.1–2.5), moderate (2.6–5), significant (5.1–7.5), high (7.6–10), and severe (> 10)

The trend of the Clean Coast Index (CCI) was analyzed on Bocagrande Beach over a 5-year monitoring period, with results presented in Fig. 3a. Between 2011 and 2015, the beach was, on average, classified within the “dirty” category, exhibiting considerable variability in CCI values over time. Notable peaks were observed in May, June, August, and September, reaching levels indicative of extremely dirty conditions. This pattern is likely associated with seasonal increases in visitor numbers during peak tourism months, combined with favorable weather conditions such as sunny days that encourage higher beach attendance.

A comparison was established between the results obtained at Bocagrande Beach and the 24 beaches monitored by Rangel-Buitrago et al. (2021) located in the northern Caribbean region of Colombia. In the latter beaches, 13% are classified as dirty, and 75% are categorized as extremely dirty. For the case of Bocagrande, the annual average was calculated during the monitoring years. In the study period from 2011 to 2014, the average CCI fluctuated between 11 and 18, categorizing the beaches as dirty. For the year 2015, an average CCI of 8 was obtained, categorizing it as a beach of moderate cleanliness. From these results, it can be inferred that during the monitoring years, the behavior of the study beach is like that reported by the Rangel-Buitrago et al. (2021) study.

Other previous studies include the one conducted in Santa Catarina, Brazil, where 17 beaches (68%) of those studied were classified, considering the overall CCI, as extremely dirty beaches (Marin et al. 2019). Additionally, in a previous study in Istanbul, Turkey, the CCI revealed that all the sample sites were categorized as extremely dirty, with CCI ranging between 29.20 and 93.40 (Akarsu et al. 2022).

The Plastic Abundance Index (PAI) values for the different monitoring years indicate that the index generally remained below 4, reflecting a moderate abundance in 59.5% of the samples. However, higher values were recorded in May and June, which, similarly to the CCI results, may be associated with peak tourist seasons on the beach (Fig. 3b). Comparative studies, such as those conducted by Rangel-Buitrago et al. (2021) on Colombian Caribbean beaches, report PAI values indicative of high plastic abundance. In contrast, beaches on Okinawa Island, Japan, exhibit PAI values ranging from low (0.1–1) to moderate (1.1–4) abundance categories (Ilechukwu et al. 2024), highlighting regional variability in plastic litter levels.

Given that plastics and CBs were the most prevalent waste items during the 2011–2015 period, the Cigarette Butt Abundance Index (CBAI) was also evaluated as a key indicator. As shown in Fig. 3c, CBAI values predominantly fell within the 1.1–4 range, corresponding to moderate abundance in 58.5% of the samples. However, due to the toxic properties of CBs, their persistence in sand and potential contribution to marine pollution warrant special attention.

To further assess the environmental impact of CBs, the Cigarette Butt Pollution Index (CBPI) was calculated based on CB density (Fig. 3d). The results indicate contamination levels ranging from low to significant throughout the 2011–2015 period, with 2015 recording the lowest CBPI value. Notably, CBPI values showed marked increases in May, June, September, and October, a seasonal trend also reflected in the CCI analysis. These patterns reinforce the link between tourist activity and increased CB-related pollution on the beach.

Other studies have reported variable CBPI values in environments comparable to beach settings, such as those recorded in Morocco in 2021, with CBPI values ranging from 0.27 to 13.51 (Mghili et al. 2023); in beaches and urban areas along the southern coast of the Caspian Sea (2021), with values between 1.20 and 27.32 (Yousefi Nasab et al. 2022); and in Bangladesh at Cox's Bazar Sea Beach (2023), with a CBPI value of 7.78 (Howlader et al. 2023).

### CB density on Bocagrande Beach during the 2021–2022 monitoring campaign

**Fig. 4:**
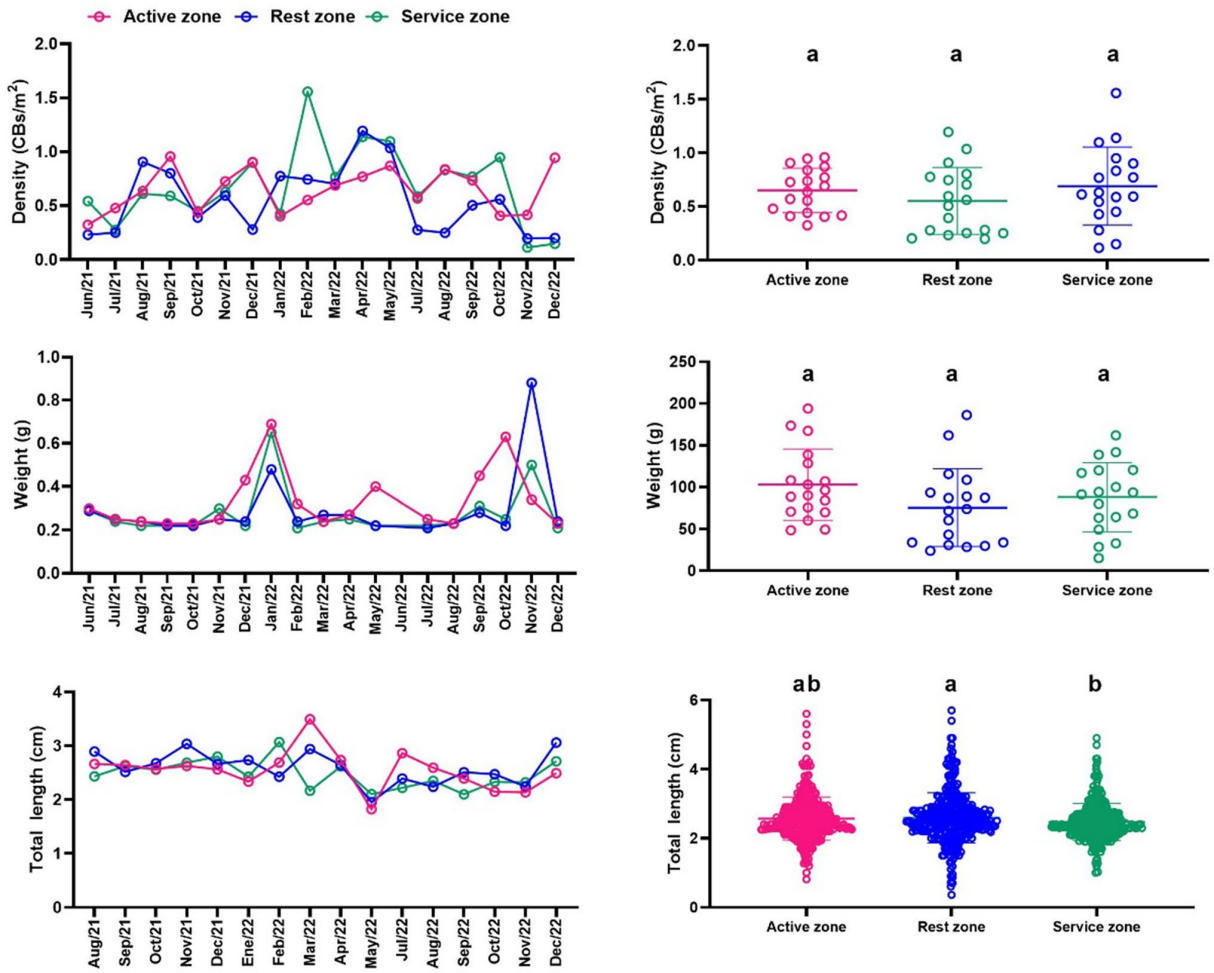
Density, weight, and total length of CBs 2021–2022. Different letters among the beach zones indicate significant differences with *p*-value < 0.05

The density of CBs per zone reached an average of approximately 0.6 CBs/m^2^, with recorded maximum and minimum values of 1.56 and 0.15 CBs/m^2^, respectively, as illustrated in Fig. 4. These values represent a substantial increase compared to the average density of 0.19 CBs/m^2^ previously reported for the same beach during the 2011–2015 period, as detailed in “Assessment of beach litter data from the 2011–2015 monitoring period at the study site” section. The behavior in the service area is highlighted, where the highest abundances are observed, possibly related to the commercial nature of this area, which involves the sale and consumption of food, beverages, and cigarettes.

In Fig. 4, it can also be observed that when comparing the three beach zones in general, significant differences (*p*-value = 0.037) are only present in the parameter"Length of cigarette butts"between the rest area and the services area, with this value being higher in the rest area.

In previous studies, average density figures of CB in southern Spain, covering cities like Alicante, Cádiz, and Ceuta during the period from 2018 to 2019, have been documented, with average values recorded at 0.038 CBs/m^2^ (Asensio-Montesinos et al. 2021). In the province of Mazandaran, Iran, south of the Caspian Sea, the reported density was 0.106 CBs/m^2^ (Yousefi Nasab et al. 2022). At Navegantes Guarujá Beach (Brazil), the density was reported at 0.755 ± 0.32 CBs/m^2^ (Ribeiro et al. 2021a, b), while at Santos Beach (Brazil), the reported density was 0.283 ± 0.112 CBs/m^2^ (Ribeiro et al. 2021a, b). In 29 beaches along the Baltic Sea coast in Germany and Lithuania (2011–2018), using OSPAR methodology, density variation ranged between 0 and 0.018 CBs/m^2^ (Kataržytė et al., 2020). In the Persian Gulf, abundance is reported between 10.78 ± 0.33 and 9.33 ± 11.04 CBs/m^2^ (Dobaradaran et al. 2018), while at Cox's Bazar Marine Beach in Bangladesh, the reported average density was 0.388 CBs/m^2^ with ranges between 0.195 and 0.689 CBs/m^2^ (Howlader et al. 2023).

Research conducted on Saint Martin Island, the only coral island in Bangladesh and a protected area, utilized a visual survey method across three distinct use zones. Data collection occurred from 9 am to 5 pm during the peak season in December 2023. A total of 4481 CBs were recorded, with densities ranging from 0.37 to 1.76 m^2^ and an average density of 0.99 m^2^ across 12 sampling campaigns. The service zones exhibited the highest density of CBs (Howlader et al. 2024a, b). Observations from the beaches of Sonadia Island, Bangladesh, revealed 524 CBs, representing 3.54% of the total waste collected, with a recorded density of 0.025 items/m^2^ (Howlader et al. 2024a, b). A study conducted on urban beaches in the Brazilian Northeast evaluated CBs in comparison to other types of litter. Sampling took place during the high season (January 2016) at eight beaches with heavy visitor activity. CBs were classified as an isolated litter category, with a total of 10,880 items recorded, of which 38.36% were CBs (Araújo and Costa 2021). Supplementary Table 1 summarizes the average density values of CBs reported for beaches in various geographical locations, as documented in multiple scientific studies.

The comparative analysis of CB density across different beach environments reveals notable variability, with Cartagena Beach exhibiting higher density values than those reported in Spain, Iran, and Brazil. This highlights the persistent presence of this pollutant since the initial evaluation period (2011–2015) and underscores the urgent need for enhanced environmental education strategies aimed at preventing the improper disposal of cigarette butts, given their significant associated pollutant load.

The average mass of CBs found during the study period was 0.3 g, with maximum values of 0.8 g and minimum values of 0.2 g. The average value is slightly higher than the 0.17 g per filter estimated by Novotny and Slaughter (2014), which reports that the approximate weight of 20 cigarette filters is 3.4 g. According to the literature, the average weight of tobacco in a typical manufactured cigarette is approximately 0.75 g (Zafeiridou et al. 2018). However, it is important to note that the conditions under which CB samples are recovered vary due to the diversity of cigarette brands and the condition of the butts, whether fully smoked or containing residual tobacco. This variability is evident in the differing lengths of the collected CBs.

### CBF density on Bocagrande Beach during the 2021–2022 monitoring campaign

**Fig. 5:**
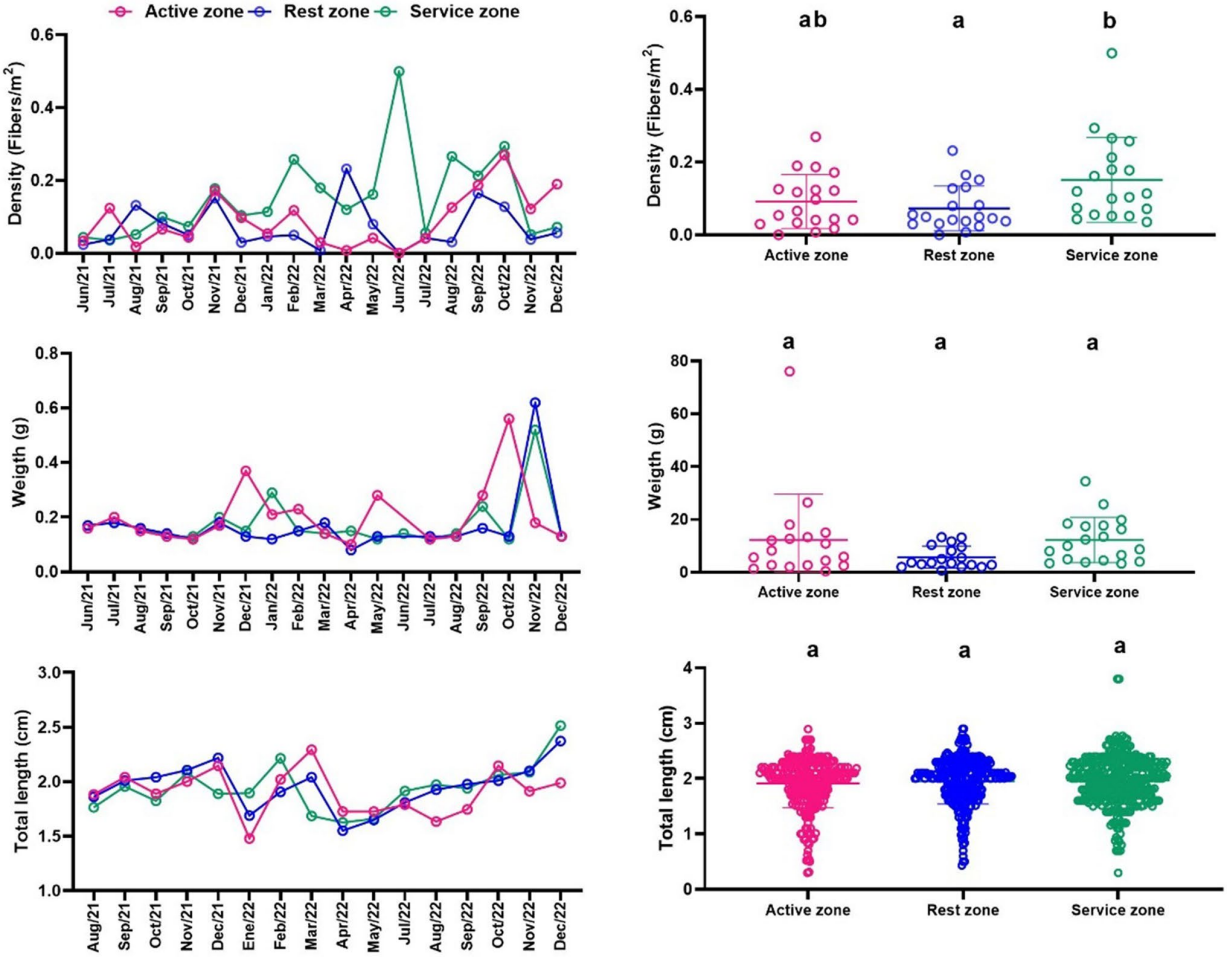
Density, weight, and total length of CBFs 2021–2022. Different letters among the beach zones indicate significant differences with *p*-value < 0.05

The spatial distribution of CBFs revealed average densities of approximately 0.1 CBFs/m^2^, with recorded maximum values of 0.27 CBFs/m^2^ and minimum values of 0.03 CBFs/m^2^ (Fig. 5). Overall, CBF densities were lower than those observed for intact cigarette butts during the same monitoring period. The highest concentrations of CBFs were found in the service zone, indicating a potential accumulation pattern associated with specific human activities. As CBFs result from the physical and chemical degradation of cigarette butts, their abundance may be influenced by environmental variables such as ambient temperature and sand granulometry. Additionally, their longer exposure to beach conditions likely contributes to mass loss and progressive fragmentation, reflecting their persistence and transformation within the coastal environment.

In the ANOVA results shown in Fig. 5, it is observed that the only parameter that showed significant differences between the beach zones was fiber density, with a *p*-value of 0.02. The density of these residues was higher in the service area.

The conditions under which CBFs are recovered indicate a mass loss from their possible initial conditions, with percentage values of mass loss ranging between 30 and 60% of the maximum and minimum values, respectively, of the CBs recovered during monitoring campaigns. The lengths of CBFs range from 1.5 to 2.5 cm. This highlights the need for further research to identify the decomposition times of CBs into CBFs related to different environments where cigarette butts are discarded. This will enable the prediction of plasticizer loss from cellulose acetate films during aging, as well as processes involving acetic acid release and plasticizer migration (Liu et al. 2019).

### Cigarette butt pollution index

**Fig. 6:**
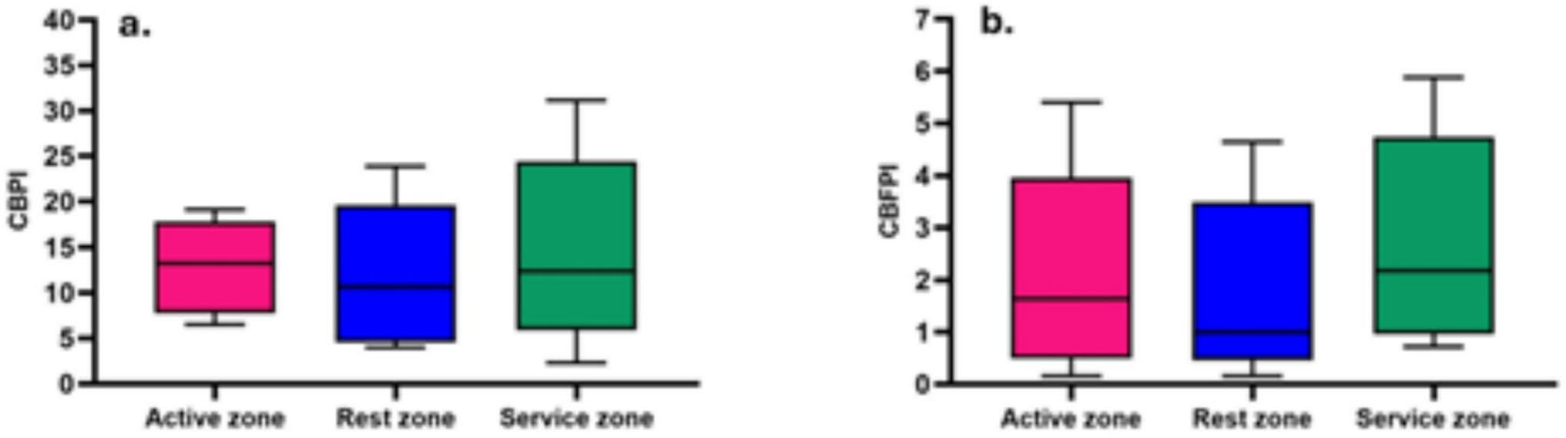
Spatial variation of the Cigarette Butt Pollution Index (CBPI) on Bocagrande Beach, Cartagena. **a** CBPI: very low (≤ 1), low (1.1–2.5), moderate (2.6–5), significant (5.1–7.5), high (7.6–10), and severe (> 10). **b** CBFPI: very low (≤ 1), low (1.1–2.5), moderate (2.6–5), significant (5.1–7.5), high (7.6–10), and severe (> 10)

To evaluate pollution levels associated with coastal litter, several quantitative indexes are applied, including targeted tools such as the Cigarette Butt Pollution Index (CBPI). The latter provides a spatial assessment of environmental conditions by measuring litter density within defined zones (Delavari Heravi et al. 2024). In this study, CBPI values were computed using data collected from June 2021 to December 2022, based on CB densities recorded across different beach-use zones (Supplementary Table 2). The resulting CBPI values ranged from 2.28 to 31.16, indicating pollution levels classified predominantly as severe with respect to cigarette butt contamination (Fig. 6a).

Results presented by Mghili et al. (2023) on the beaches of Morocco indicate variable pollution ranging from very low to severe, with a maximum reported CBPI of 35.9. They also report that CBPI values were higher during summer and winter, significantly associated with beach user density levels. For three beaches in Iran, CBPI values of 8.16, 15.68, and 27.32 were reported, also showing contamination values ranging from high to severe (Yousefi Nasab et al. 2022). This, compared to the current results obtained on the study beach in Bocagrande (Cartagena, Colombia), also showing pollution levels varying between high and severe in most months of the study, highlights the significant impact of cigarette butt contamination on the beach sand.

Table 1 summarizes studies reporting CBPI on various beaches across different geographical regions, highlighting variations in beach categorization ranging from very low pollution to severe pollution. The increasing number of studies reporting CBPI in recent years reflects the growing interest in investigating these discarded residues on beaches.

**Table 1:** CBPI research conducted on beaches worldwide

Study beach location	CBPI	CBPI status	Reference
Vung Tau beaches (Vietnam)	0.44–2.40	Very low to low pollution	(Nguyen et al. 2025)
Eleven sandy beaches located in the northwest of Morocco	0.0–35.9	Very low to severe pollution	(Mghili et al. 2023)
Cox’s Bazar Beach (southeastern coastal area of Bangladesh)	3.89–13.78	Pollution to severe pollution	(Howlader et al. 2023)
Coastal city south of the Caspian Sea in Mazandaran Province, Iran	8.16–27.32	High pollution to severe pollution	(Yousefi Nasab et al. 2022)
Eight beaches on the Yellow Sea (northwestern Pacific Ocean)	Average 10.38	Severe pollution	(Lian et al. 2024)
Fifteen sandy beaches in East Java Province, Indonesia	1.6–29.6	Low pollution to severe pollution	(Yona et al. 2024)
Five touristic beaches in Latin America	0.1–15.0	Very low pollution to severe pollution	(Díaz-Mendoza et al. 2023)
Perequê Beach (Brazil)	30.10 ± 47.50 [0.00–200.00]	Severe pollution	(Ribeiro et al. 2024)
Saint Martin Island (Bangladesh)	7.44–28.84	High pollution to severe pollution	(Howlader et al. 2024a, b)
Bocagrande Beach (Cartagena, Colombia)	2.28–31.16	Pollution to severe pollution	In this study

Additionally, the Cigarette Butt Fiber Pollution Index (CBFPI) was calculated using Eq. (4), following the same approach as for CBPI. This consideration is based on the fact that CBFs are a byproduct of the decomposition of CBs and, like CBs, are recognized as hazardous waste in beach environments. Supplementary Table 3 presents the results of CBF densities and the corresponding CBFPI values obtained during the study period. The results show values between pollution and low pollution are identified. It is important to consider that, although CBFs are less abundant and therefore report lower pollution index values, an important aspect should be reflected upon since CBFs are a byproduct of cigarette butts. As a byproduct, CBFs contain residuals of the initial pollution, which vary considering decomposition times and mechanisms. The CBFPI ranged between 0.01 and 5.88. The CBFPI results show category variations ranging from low pollution to considerable pollution, as shown in Fig. 6b.

CBs contain thousands of toxic compounds, including nicotine, tar, and heavy metals. Over 90% of modern cigarettes have single-use plastic filters made of cellulose acetate fibers and additives. As hazardous waste, CBs pose serious environmental and human health risks, serving as a pathway for toxic substances to enter aquatic ecosystems (Thuan et al. 2024). Cigarette filters are generally composed of more than 15,000 fiber strands made of cellulose acetate with plasticized additives (Belzagui et al. 2021).

The calculation of CBFPI is crucial due to the hazardous nature of CBFs in the environment. The results indicate that, although the index ranges from low to considerable contamination, the potential transformation of CB fibers into microplastics must be considered, along with their capacity to facilitate pollutant dispersion in the environment. CBFPI was reported similarly manner to this study in research conducted on five pilot beaches across Latin America, including Ecuador, Brazil, Argentina, Mexico, and Colombia. The highest CBFPI value was recorded at Peró Beach in Brazil, ranging from 1.0 (very low contamination) to 8.3 (high contamination) (Díaz-Mendoza et al. 2023).

### Correlation between environmental parameters, abundance, and density of CBs and CBFs on the study beach in Cartagena during 2021–2022

**Fig. 7:**
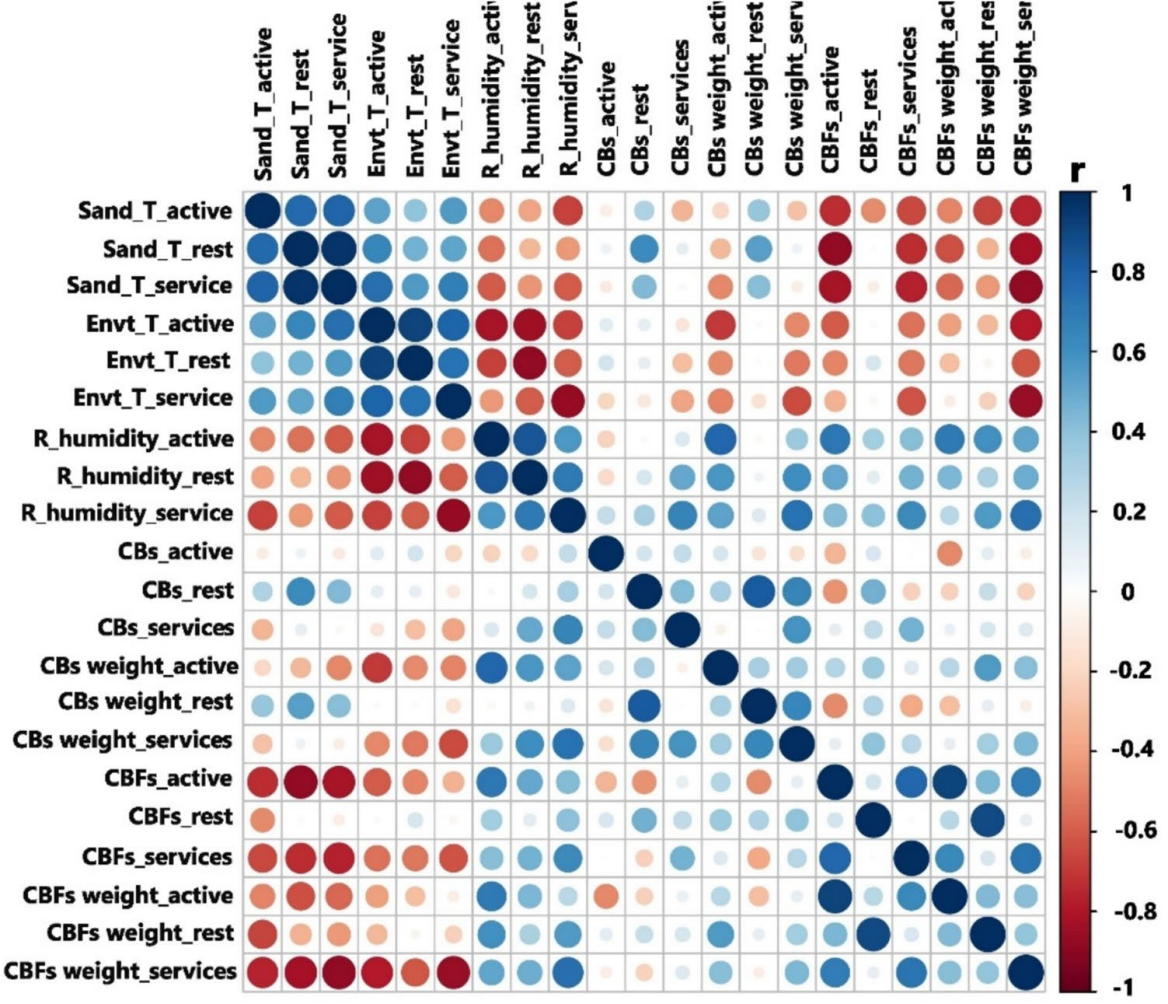
Statistical correlation of environmental parameters, abundances, and masses of CBs–CBFs on the study beach

A statistical correlation was performed between the environmental parameters of ambient temperature, sand temperature, relative humidity, and the abundance of CBs and CBFs, as well as the relationship with the average masses reported for CBs and CBFs by usage zones. Figure 7 shows that both positive and negative correlations can be obtained with high significance.

Figure 7 demonstrates a positive statistical correlation between the abundance and mass of CBs and temperature in the rest area, which leads to lower moisture levels in the sand of this zone. This could suggest that the CBs collected in this area are more likely to have been recently discarded, as they have not yet undergone their initial decomposition process and, therefore, have retained their original filter wrapper. Additionally, Fig. 7 shows a correlation between the abundance and mass of CBFs and relative humidity in the active zone, which could be attributed to the moisture conditions of the sand and its proximity to the sea.

The most significant negative correlation is observed between sand temperature in the services area and the mass of fibers found in that zone. This can be explained by the fact that an increase in sand temperature in the services area leads to decreased fiber mass. This finding suggests that one of the factors influencing the mass loss processes of cellulose acetate could be both sand and ambient temperatures. In alignment with the obtained results, the literature reports that factors affecting the decomposition rate of cellulose acetate fibers in CBs can be extrinsic, such as environmental conditions, or intrinsic, including chemical composition, crystallinity, plasticizers used, and the surface characteristics of cellulose acetate—factors that must be considered (Marques et al. 2024). It is important to consider in studies on mass loss temperatures, as in the case of cellulose acetate, that the decomposition temperature indicates the breakdown of the polymer into smaller molecules and constituent atoms (Silva et al. 2023).

The study conducted by Bonanomi et al. (2020) examined the decomposition of CBs over a 5-year period, establishing a relationship between nitrogen (N) availability, microbiome composition, and the breakdown of CBs in grassland soils. The findings suggest that under these conditions, decomposition reaches approximately 80%, whereas in dune sand, it ranges around 70%. Overall, this indicates that the decomposition process is slower in beach sand compared to grassland areas, likely due to the nitrogen dynamics in grasslands, which promote decomposition.

The study conducted by Wang et al. (2012) demonstrated a decreasing trend in NO₂ absorption in the soil as relative humidity increased from 5 to 80%. Regarding the effect of temperature, the initial NO₂ absorption coefficients declined as the temperature rose from 4.8 to 54.8°C. This finding is relevant when comparing it to the results obtained in the present study, where relative humidity, ambient temperature, and sand temperature in the services area were high, averaging 70.4%, 32.6°C, and 41.3°C, respectively. This area exhibited a higher abundance of CBFs, which could indicate an increased decomposition process of CBs into CBFs in this zone.

## Conclusions

The tourist beach of Bocagrande in Cartagena (Colombia) shows a persistent problem of inadequate litter management, especially regarding plastics and CBs. From 2011 to 2015, the average solid waste density was 0.63 items/m^2^, with plastics and CBs accounting for 0.16 and 0.19 items/m^2^, respectively. In 2021–2022, CB density increased notably to an average of 0.6 CBs/m^2^ (range: 0.15–1.56 CBs/m^2^). The Clean Coast Index initially classified the beach between moderate and dirty, while the Plastic and Cigarette Butt Abundance Indices indicated moderate pollution. The Cigarette Butt Pollution Index in the second campaign ranged from moderate to severe, evidencing worsening CB contamination. These results emphasize the urgent need for improved litter management and policy review, with a focus on environmental education to reduce CB pollution. Additionally, the findings highlight future research opportunities related to the degradation of CB fibers, heavy metal content, and microplastic formation from cellulose acetate. Including user density data in future monitoring could enhance understanding of the relationship between tourism and litter accumulation. The elevated CB and CBF pollution indices reinforce the need for targeted mitigation strategies to address this ongoing environmental challenge in coastal urban areas.

## Supplementary Information

Below is the link to the electronic supplementary material.

Supplementary file1 (DOCX 375 KB)

## Data Availability

The data will be available on request.
