# Supplementary material for: Temporal analysis of cigarette butt accumulation on a touristic beach in Cartagena, Colombia
Source: Environ Sci Pollut Res Int. 2025 Aug 6;32(33):19712–24. doi: 10.1007/s11356-025-36752-2 (PMC12426091; doi:10.1007/s11356-025-36752-2)
Supplement: Supplementary file 1 — Supplementary file1 (DOCX 375 KB) [file 11356_2025_36752_MOESM1_ESM.docx]

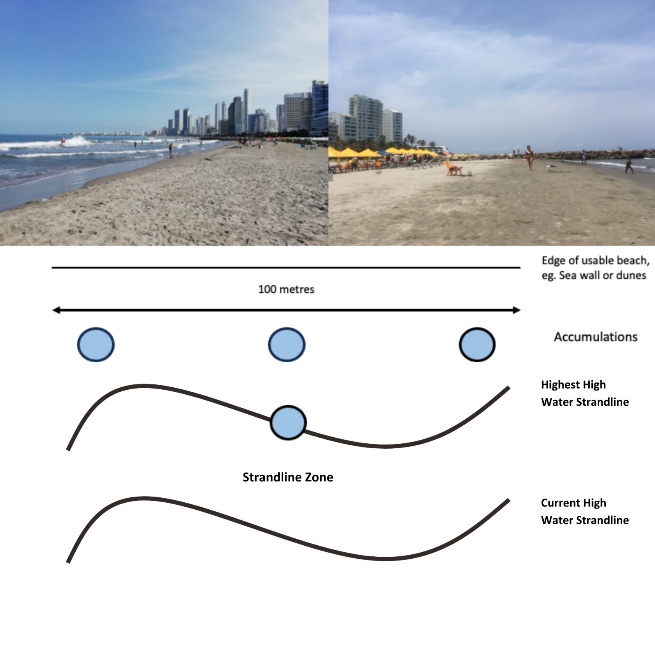


**Supplementary Figure 1.** Schematic representation of the litter collection methodology in the active zone of Bocagrande Beach, Cartagena, Colombia.


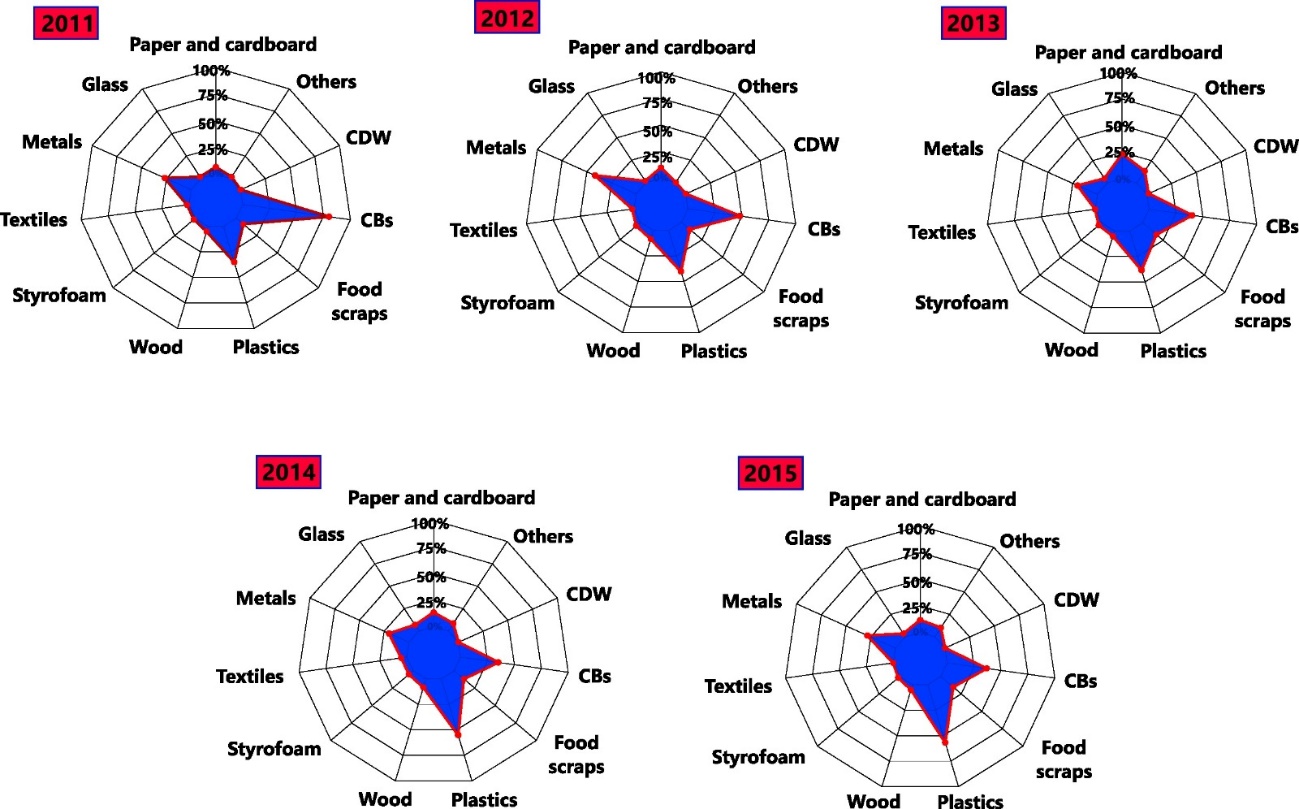


**Supplementary Figure 2.** Percentage distribution of solid waste on the sand of Bocagrande Beach

**Supplementary Table 1.** Density of CBs in different beaches around the world

| Study beach location | Density (CBs/m^2^) | Reference |
| --- | --- | --- |
| Vung Tau beaches (Vietnam) | average density of 0.085 ± 0.025 | (Nguyen et al., 2025) |
| Eleven sandy beaches located in the northwest of Morocco | average density of 0.06 | (Mghili et al., 2023) |
| Cox’s Bazar Beach (southeastern coastal area of Bangladesh) | mean density of 0.388 | (Howlader et al., 2023a) |
| Coastal city south of the Caspian Sea in Mazandaran Province, Iran | average density of 0.106 | (Nasab et al., 2022) |
| Eight beaches on the Yellow Sea (northwestern Pacific Ocean) | average density of 0.23 | (Lian et al., 2024) |
| Fifteen sandy beaches in East Java Province, Indonesia | densities ranging from 0.08 to 3.32 | (Yona et al., 2024) |
| Five touristic beaches in Latin America | average density of 0.005-0.750 | (Díaz-Mendoza et al., 2023) |
| Perequˆe beach (Brazil) | average density 0.15 ± 0.01 | (Ribeiro et al., 2024) |
| Saint Martin Island (Bangladesh) | mean density of 0.99 | (Howlader, Selim, et al., 2024) |
| The seacoast of Mazandaran Province (Iran) | average 1.4028 ± 1.2328 | (Kouhi et al., 2025) |
| 29 beaches on the German and Lithuanian Baltic Sea coast | Mean: 0.018 ±0.07 – 0.18 ±0.403 | (Kataržytė et al., 2020) |
| Two popular public beaches in Thailand | mean butt density of 0.44 | (Kungskulniti et al., 2018) |
| Beaches located in the left margin of Santos Estuarine System (Guarujá City - Brazil) | densities ranging from 0.755 ± 0.32; 0.078 ± 0.02; 0.041 ± 0.01 | (Ribeiro, Harayashiki, et al., 2021b) |
| Mesotidal coast of Cádiz Province and the microtidal coasts of Alacant and Ceuta (Spanish) | densities ranging from 0.033–0.342 | (Asensio-Montesinos et al., 2021a) |
| Dalian on the southern part of the Liaodong Peninsula in Liaoning Province, Northeast China | 0.059 ± 0.002  in workdays, and 0.094 ± 0.003  in weekends | (Yang et al., 2023) |
| Bocagrande Beach (Cartagena, Colombia) | densities ranging from 0.11 - 1.56 | In this study |

**Supplementary Table 2.** CBs density and CBPI according to the usage zone in the study beach

| Date | Active Zone | | Rest Zone | | Service Zone | |
| --- | --- | --- | --- | --- | --- | --- |
|  | CBs/m^2^ | CBPI | CBs/m^2^ | CBPI | CBs/m^2^ | CBPI |
| June-21 | 0.32 | 6.48 | 0.23 | 4.64 | 0.54 | 10.88 |
| July-21 | 0.48 | 9.60 | 0.25 | 5.04 | 0.28 | 5.56 |
| August-21 | 0.64 | 12.76 | 0.91 | 18.16 | 0.61 | 12.24 |
| September-21 | 0.96 | 19.16 | 0.80 | 16.04 | 0.59 | 11.84 |
| October-21 | 0.44 | 8.80 | 0.39 | 7.84 | 0.45 | 9.00 |
| November-21 | 0.73 | 14.56 | 0.60 | 11.92 | 0.63 | 12.60 |
| December-21 | 0.91 | 18.12 | 0.28 | 5.60 | 0.90 | 18.00 |
| January-22 | 0.40 | 8.08 | 0.78 | 15.52 | 0.43 | 8.56 |
| February-22 | 0.55 | 11.08 | 0.75 | 14.92 | 1.56 | 31.16 |
| March-22 | 0.69 | 13.80 | 0.70 | 14.08 | 0.77 | 15.36 |
| April-22 | 0.77 | 15.40 | 1.19 | 23.88 | 1.14 | 22.80 |
| May-22 | 0.87 | 17.40 | 1.04 | 20.72 | 1.10 | 21.96 |
| July-22 | 0.57 | 11.38 | 0.28 | 5.54 | 0.59 | 11.74 |
| August-22 | 0.84 | 16.80 | 0.25 | 5.00 | 0.83 | 16.62 |
| September-22 | 0.74 | 14.76 | 0.51 | 10.10 | 0.77 | 15.38 |
| October-22 | 0.41 | 8.20 | 0.56 | 11.20 | 0.95 | 19.00 |
| November-22 | 0.42 | 8.32 | 0.20 | 3.96 | 0.11 | 2.28 |
| December-22 | 0.95 | 18.92 | 0.20 | 4.04 | 0.15 | 3.00 |

**Supplementary Table 3.** CBFs density -CBFPI according to the usage zone in the study beach

| Date | Active Zone | | Rest Zone | | Service Zone | |
| --- | --- | --- | --- | --- | --- | --- |
|  | CBFs/m^2^ | CBFPI | CBFs/m^2^ | CBFPI | CBFs/m^2^ | CBFPI |
| June-21 | 0.03 | 0.68 | 0.02 | 0.48 | 0.04 | 0.88 |
| July-21 | 0.12 | 2.48 | 0.04 | 0.76 | 0.04 | 0.72 |
| August-21 | 0.02 | 0.36 | 0.13 | 2.64 | 0.05 | 1.04 |
| September-21 | 0.07 | 1.32 | 0.08 | 1.64 | 0.10 | 2.00 |
| October-21 | 0.04 | 0.88 | 0.05 | 1.00 | 0.07 | 1.48 |
| November-21 | 0.17 | 3.44 | 0.15 | 3.04 | 0.18 | 3.56 |
| December-21 | 0.10 | 1.96 | 0.03 | 0.60 | 0.10 | 2.08 |
| January-22 | 0.05 | 1.08 | 0.05 | 0.92 | 0.11 | 2.28 |
| February-22 | 0.12 | 2.36 | 0.05 | 1.00 | 0.26 | 5.16 |
| March-22 | 0.03 | 0.60 | 0.01 | 0.16 | 0.18 | 3.60 |
| April-22 | 0.01 | 0.16 | 0.23 | 4.64 | 0.12 | 2.40 |
| May-22 | 0.04 | 0.84 | 0.08 | 1.60 | 0.16 | 3.24 |
| July-22 | 0.04 | 0.82 | 0.04 | 0.84 | 0.06 | 1.12 |
| August-22 | 0.13 | 2.52 | 0.03 | 0.62 | 0.27 | 5.32 |
| September-22 | 0.19 | 3.74 | 0.17 | 3.30 | 0.21 | 4.26 |
| October-22 | 0.27 | 5.40 | 0.13 | 2.56 | 0.29 | 5.88 |
| November-22 | 0.12 | 2.44 | 0.04 | 0.76 | 0.05 | 1.04 |
| December-22 | 0.19 | 3.80 | 0.06 | 1.12 | 0.07 | 1.44 |
